# Comparison of four-dimensional CT and Sestamibi SPECTCT in the localization management of primary hyperparathyroidism

**DOI:** 10.1186/s40644-025-00897-7

**Published:** 2025-07-11

**Authors:** Jun Yang, Xili Lu, Pingping Zhou, Zhonghui Gao, Cheng Ding, Wanwen Weng, Linpeng Yao, Xinhui Su

**Affiliations:** 1 Department of Nuclear Medicine, the First Affiliated Hospital, College of Medicine, Zhejiang University, 310003, #79 Qingchun Road, Hangzhou, 310003 P.R. China; 2 The Department of Radiology, Xiangshan First People’s Hospital Medical and Health Group, #291 Donggu Road, Dandong Street, Ningbo, 315700 P.R. China; 3 State Key Laboratory for Diagnosis and Treatment of Infectious Diseases, National Clinical Research Center for Infectious Diseases, the First Affiliated Hospital, Zhejiang University School of Medicine, 310003, #79 Qingchu Road, Hangzhou, P.R. China; 4 The Department of Radiology, the First Affiliated Hospital, College of Medicine, Zhejiang University, 310003, #79 Qingchun Road, Hangzhou, P.R. China

**Keywords:** 4D-CT, Sestamibi, SPECT/CT, Primary hyperparathyroidism

## Abstract

**Objective:**

Accurate preoperative imaging localization is paramount to the success of targeted parathyroidectomy for primary hyperparathyroidism (PHPT). Four-dimensional (4D) CT is a promising method for preoperative localization of the parathyroid, but studies on the performance of 4D CT and technetium 99 m-sestamibi SPECT/CT for the diagnosis of diseases of the parathyroid are limited.

**Materials and methods:**

To compare the diagnostic performance of sestamibi SPECT/CT and 4D-CT for preoperative localization in patients with PHPT in a single-institution from August 2017 to May 2024.

**Results:**

Two hundred forty-two patients with PHPT (166 females; 52.5 years ± 13.4 [SD]) were evaluated. Among the 242 patients, 233 patients (96.3%) had single-gland disease, and 9 patients (3.7%) had multigland disease. Similar diagnostic performance was observed for sestamibi SPECT/CT and 4D-CT ([receiver operating characteristic ROC], 0.90 [95% CI: 0.87, 0.92] and 0.88 [95% CI: 0.85, 0.90], respectively; *p* = 0.11). Compared with 4D-CT, combined-modality sensitive reading and sestamibi SPECT/CT had the highest ROC, and, although there was no significant difference between the two (ROC, 0.91; 95% CI: 0.89, 0.93; *p* = 0.14), they significantly differed from 4D-CT (*p* = 0.0006). Sestamibi SPECT/CT showed an accuracy of 92% (95% CI: 90%, 94%), similar to 4D-CT (91%; 95% CI: 89%, 92%), combined-modality sensitive reading (91%; 95% CI: 89%, 93%) and combined-modality specificity reading (92%; 95% CI: 90%, 94%).

**Conclusion:**

Sestamibi SPECT/CT has high accuracy in preoperative localization in patients with PHPT. Compared with sestamibi SPECT/CT alone, 4D-CT and combined-modality reading did not improve diagnostic performance.

## Introduction

Primary hyperparathyroidism (PHPT) is one of the most common endocrine disorders, with a prevalence of approximately 1%; notably, the prevalence of PHPT has increased in recent decades due to routine biological screening [1]. Diagnosis is based on elevated serum calcium levels, low phosphorus levels, and abnormal parathyroid hormone (PTH) levels [2]. More than 90% of patients with PHPT have a single parathyroid adenoma, and, in rare cases, PHPT is associated with multiglandular hyperplasia [2, 3]. Surgery is a definitive curative treatment; however, the outcomes of other treatment schemes for PHPT, including bilateral neck exploration followed by targeted parathyroidectomy, have been improved owing to improvements in preoperative imaging, minimally invasive technology, and intraoperative monitoring of PTH [3]. Accurate preoperative localization of the abnormal parathyroid gland is critical to the success of parathyroidectomy because it reduces complication rates, shortens the operation time, improves cosmetic outcomes, accelerates recovery, reduces overall costs, and improves cure rates [4].

Because several imaging modalities with different acquisition protocols are available for localization, there is no current consensus on the optimal preoperative imaging modality. Currently, the modality is selected on the basis of the expertise of both the surgeon and the radiologist as well as the capabilities of each imaging modality. Ultrasonography and/or sestamibi SPECT/CT are commonly employed imaging methods for preoperative localization owing to their good sensitivity and positive predictive value in most clinical settings [5]. Since 2006, four-dimensional (4D) CT has been used as a promising method for the imaging of the parathyroid as it is highly accurate and cost-effective [6]. Compared with ultrasonography and sestamibi SPECT/CT, 4D-CT is reported to be more accurate and sensitive, especially for patients with negative or inconclusive findings on ultrasonography and sestamibi examinations, patients who are exhibiting signs of recurrence or persistent hyperthyroidism after parathyroidectomy or previous neck surgery, and patients with multigland disease [7–10]. However, lesions can still be missed on 4D-CT images, especially in cases of multigland disease, small parathyroid lesions, thyroid nodules and parathyroid lesions positioned inferiorly [11]. Therefore, rigorous head-to-head comparisons of 4D-CT and sestamibi SPECT/CT images without preselecting patients are still lacking and have been limited by the small number of study participants.

The aim of this study was to investigate whether the diagnostic performance of the combination of 4D-CT and sestamibi SPECT/CT could outperform individual imaging modalities and whether 4D-CT could outperform sestamibi SPECT/CT for preoperative localization in patients with PHPT.

## Materials and methods

### Patients

From August 2017 to May 2024, we retrospectively reviewed the clinical data of consecutive patients with laboratory test results and clinical evidence confirming the diagnosis of PHPT [3]. Patients were eligible if they underwent both of the two imaging modalities, including 4D-CT and sestamibi SPECT/CT, and underwent parathyroid surgery at our institution. The exclusion criteria were as follows: age < 18 years, previous history of thyroid or parathyroid surgery, incomplete 4D-CT or sestamibi SPECT/CT data, poor surgical outcomes, persistent hyperparathyroidism during the follow-up period, ectopic parathyroid adenoma in the mediastinum, pathologically confirmed parathyroid carcinoma and followed up for less than 1 month. The study was approved, and the requirement for written consent was waived by the institutional review board, and was compliant with the Health and Insurance Portability and Accountability Act.

### Imaging protocols

Standard dual-phase sestamibi parathyroid scintigraphy in our institution consists of early-phase planar (15 min), delayed-phase planar and SPECT/CT (120 min) methods. Images were acquired with SPECT/CT scanners (GE Medical systems, Discovery 670/670Pro; Siemens Medical Solutions, Symbia Intevo Bold) via a low-energy, high-spatial-resolution parallel hole collimator. The level of the parotid to the glands to the heart were scanned after the patient received 550 MBq of sestamibi. The scintigraphy acquisition settings and CT parameters were as follows: 64 frames over 360^0^; 20 s/projection; window matrix, 128 × 128; 120 kV; 100–440 mA by automatic exposure; slice thickness, 1.25 mm; and beam width, 2.5 mm. The planar and reconstructed SPECT/CT images were displayed and reviewed using commercial workstations (Xeleris ™, GE Healthcare; Syngo.via, Siemens Healthineers).

With the patient lying supine, the neck extended and the arms lying alongside the body, 4D-CT images, which included noncontrast, arterial phase, and delayed phase images, were acquired with a 64–256 row multirow CT detector from the mandibular angle to the tracheal carina. The scanning parameters were as follows: pitch factor, 0.6; tube rotation time, 0.6–0.8 s; 120kVp; and automatic tube current modulation. Iodinated contrast agent was injected at 3 mL/second for a total volume of 60 mL. Arterial phase images were acquired 30 s after the start of the injection, and delayed phase images were acquired 60 s after the start of the injection. The images were reconstructed with a 1.25 mm section thickness.

### Image analysis

Sestamibi SPECT/CT and 4D-CT were performed within 2 weeks to ensure comparable diagnostic conditions. All the images were reviewed and re-evaluated for localization on a workstation by two nuclear medicine specialists and radiologists. All the readers were informed that the patient was diagnosed with PHPT. Focal nonphysiological uptake consistent with expected parathyroid sites or ectopic areas with nodules was considered positive on either early- or delayed-phase image. On serial phase 4D-CT images, parathyroid nodules exhibited expected patterns of contrast enhancement. The third reader re-evaluated and settled disagreements when the two readers disagreed. After the thyroid was divided along the midline, abnormal parathyroid lesions were observed in four quadrants.

### Surgical procedures and follow-up

All patients underwent parathyroidectomy under general anesthesia within 4 weeks of the last imaging examination. The surgeon selected the surgical procedure on the basis of clinical factors and the findings of reports involving a combination of imaging modalities, such as reports on the use of color Doppler ultrasonography of the necks of patients with possible coexisting thyroid disease. The number and locations of the removed parathyroid adenoma and the incidence of pathologically confirmed hyperplasia were collected from surgical reports. The patients underwent relevant blood tests at least 1 month postoperatively. Surgical reports, pathological confirmation of parathyroid adenoma or hyperplasia and normalization of calcium levels after more than 1 month of follow-up were considered the reference standards for assessing image accuracy.

### Statistical analysis

Continuous variables are presented as means with standard deviations, and qualitative variables are presented as percentages. In the sensitive analysis, if either sestamibi SPECT/CT or 4D-CT was positive for a hyperfunctioning gland within a quadrant, this was considered to be a positive result. In the specific analysis, both sestamibi SPECT/CT and 4D-CT had to be positive to be considered a positive result. Analyses of the sensitivity and specificity of 4D-CT and sestamibi were performed to interpret the results reported in the literature [12]. The identification of adenomas and/or hyperplasia vs. normal histology was analyzed per lesion by accuracy, sensitivity, specificity, positive predictive value (PPV), and negative predictive value (NPV), according to the pathological results. The diagnostic performance of sestamibi SPECT/CT, 4D-CT and the combination of two image modules was calculated via a binary classifier system and a nonparametric receiver operating characteristic (ROC) curve. A *p* value less than 0.05 was considered statistically significant. Statistical analysis was performed using the statistical software package SPSS (PASW version 18.0 for Windows; SPSS Inc, Chicago, IL).

## Results

**Fig. 1:**
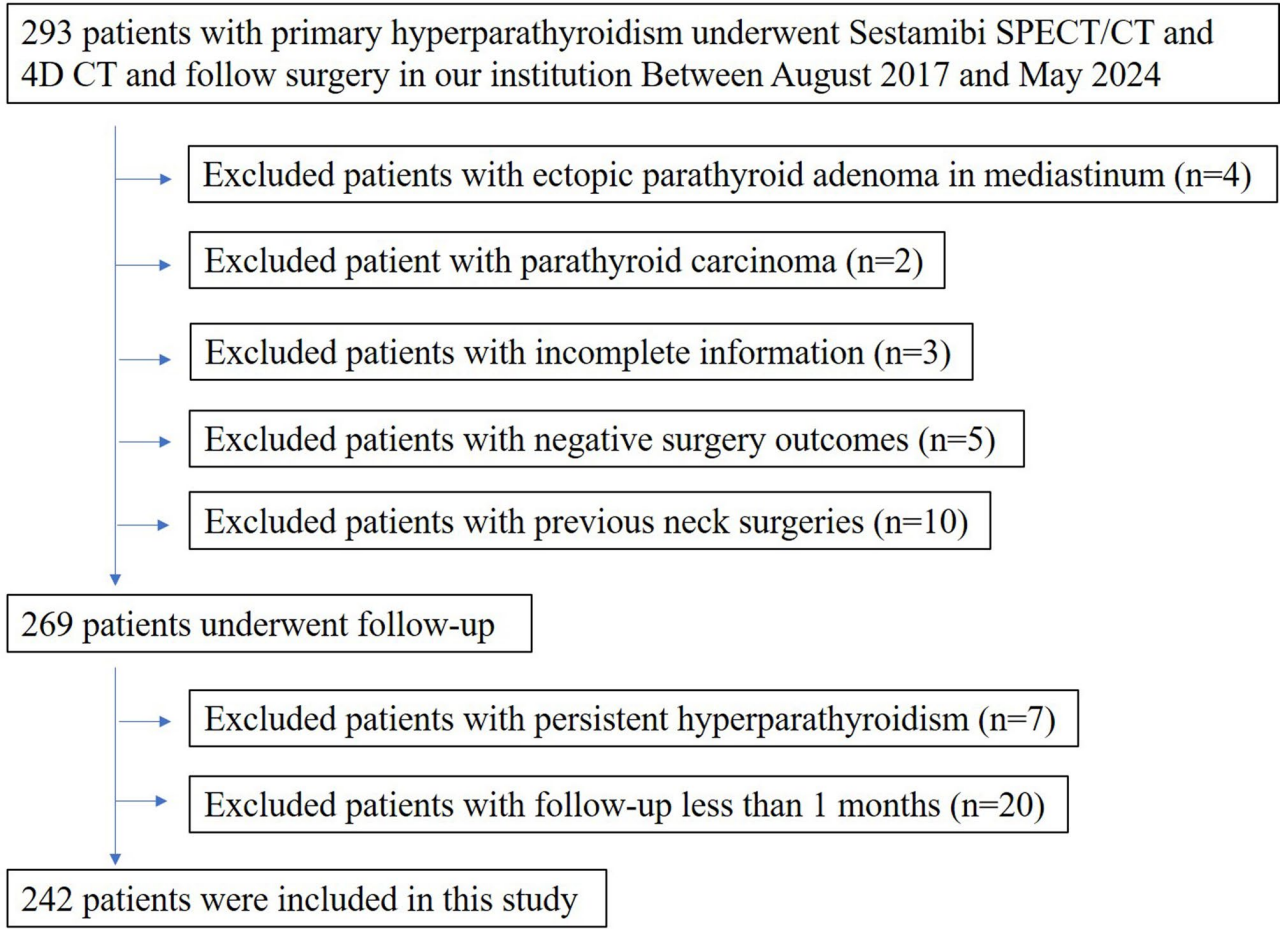
Study flowchart with consecutive patients with primary hyperparathyroidism and inclusion and exclusion criteria

Between August 2017 and May 2024, 293 patients with PHPT underwent a combination of imaging tests followed by parathyroidectomy at our institution. Fifty-one patients were excluded: 10 patients due to a history of thyroid or parathyroid surgery, 3 patients due to incomplete imaging information, 5 patients due to unfavorable surgical outcomes, 7 patients due to persistent hyperparathyroidism, 4 patients due to ectopic parathyroid adenoma, 2 patients due to parathyroid carcinoma, and 20 patients due to being followed up for less than one month. Figure 1 shows the flow of patient inclusion in our study. The clinical and pathological characteristics of the 242 patients (166 females; mean ± SD age: 52.5 ± 13.4; age range: 20–83 years) included in the final analysis are listed in Table 1. The mean calcium, phosphorus and 25-hydroxyvitamin D levels were 2.80 mmol/L (normal, 2.08–2.60), 0.87 mmol/L (normal, 0.85–1.51) and 31.6 ng/mL (normal, 12.3–107), respectively. The median PTH, alkaline phosphate and creatinine levels were 256.4 pg/mL (normal, 12–65), 134.5 U/L (normal, 45–125) and 73.4 µmol/L (normal, 41–97), respectively.

**Table 1:** Clinical and pathologic characteristics of patients with primary hyperparathyroidism

Parameter	Total (*n* = 242)
Clinical characteristics	
Age, mean (range), y	52.49 (20–83)
Female, No. (%)	166 (68.60)
BMI, mean (SD)	23.44 (3.28)
BMI missing, No. (%)	7 (2.89)
Thyroid nodule, No. (%)	100 (41.32)
Biological characteristics, mean (SD)	
Calcium, mean (SD), mmol/L	2.80 (0.32)
Phosphorus, mean (SD), mmol/L	0.87 (0.21)
25-hydroxyvitamin D, mean (range), ng/mL	31.6 (7.5–109)
25-hydroxyvitamin D, missing, No. (%)	26 (10.74)
Preoperative PTH, median (quartile), pg/mL	256.41 (149)
Alkaline phosphates, median (quartile), (U/L)	134.51 (107)
Creatinine, median (SD), (µmol/L)	73.41 (32.79)
Pathologic characteristics	
Parathyroid lesion size, mean (SD), mm	18.55 (9.24)
Single gland No. (%)	233 (96.28)
Multigland No. (%)	9 (3.72)
Location	
Left superior No. (%)	45 (17.93)
Left inferior No. (%)	75 (29.88)
Right superior No. (%)	31 (12.4)
Right inferior No. (%)	100 (39.84)
Follow up, median (range), months	6 (1–69)
Abbreviation: BMI, body mass index; PTH, parathyroid hormone; Calcium, normal range: 2.11–2.52; Phosphorus, normal range: 0.85–1.51; 25-hydroxyvitamin D, normal range: 12.3–107; PTH, normal range: 12.0–65.0; Alkaline phosphates, normal range: 45–125; Creatinine, normal range: 41–97; MGD, multigland disease.	

### Parathyroid disease characteristics and follow-up

A total of 242 patients were assumed to have four parathyroid glands for the four-quadrant analysis (*n* = 968). Among the 242 patients, 233 patients (96.3%) had single-gland disease, and 9 patients (3.7%) had multigland disease. All 9 patients with multigland disease had double adenomas or hyperplasia. The 251 lesions had a mean (± SD) maximal diameter at pathologic evaluation of 18.6 mm ± 9.2. A total of 120 of 251 (47.8%) were from the left, and 131 of 251 (52.2%) were from the right. The distribution of abnormal glands per quadrant is summarized in Table 1. All patients were followed up for at least one month. The median follow-up time was 6 months (range 1–69 months).

### Diagnostic performance

**Fig. 2:**
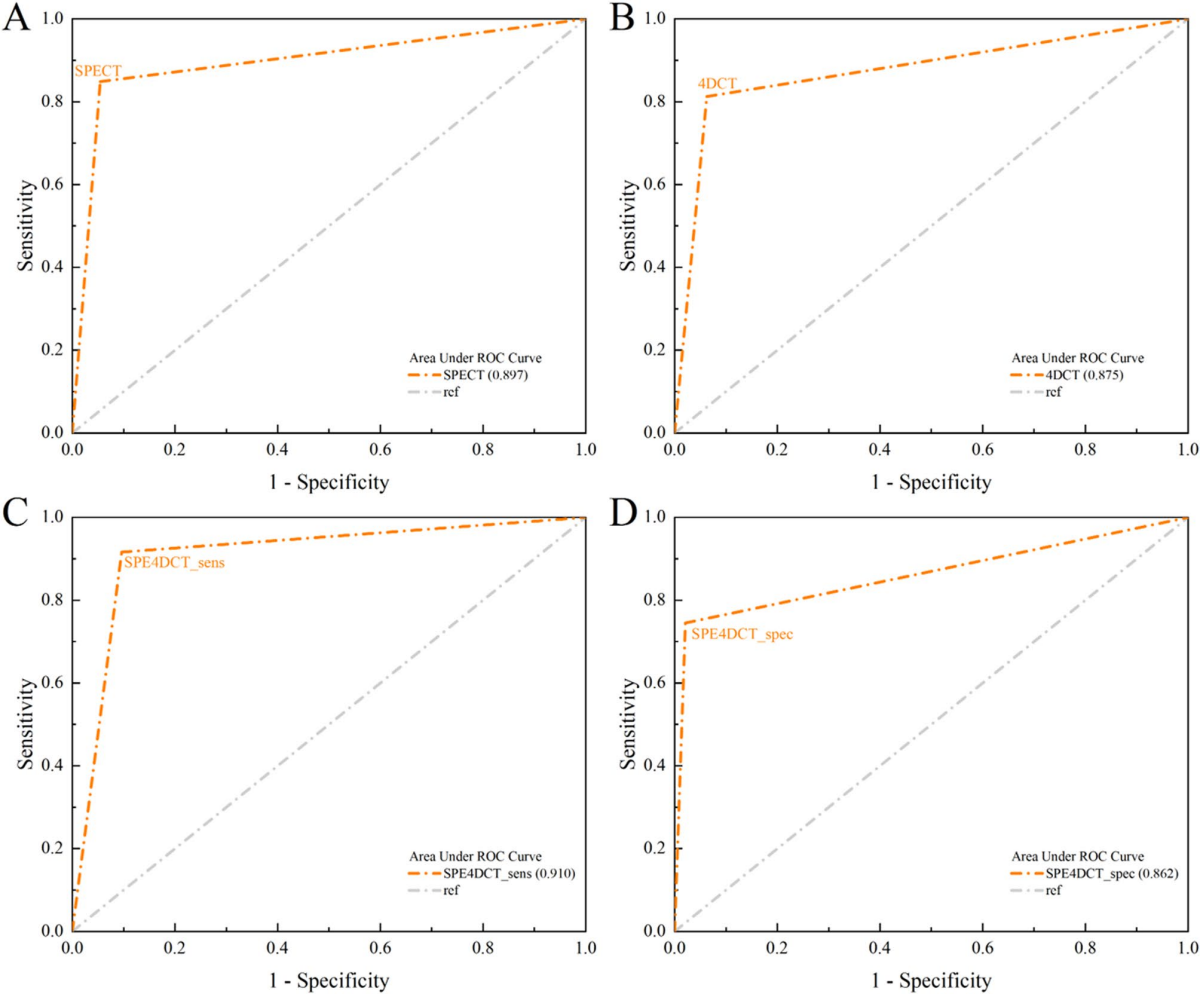
Receiver operating characteristic (ROC) curves for sestamibi SPEC/TC (**A**), 4D-CT (**B**), combined 4D-CT with Sestamibi SPECT/CT sensitive reading (**C**), and combined 4D-CT with Sestamibi SPECT/CT specific reading (**D**) via a per-lesion, four-quadrant analysis

In 242 patients, 768 parathyroid glands were normal or abnormal according to the per quadrant analysis for the diagnostic performance of each imaging modality. Figure 2; Table 2 show the ROCs for all the modalities and a comparison of the ROCs for both sestamibi SPECT/CT and 4D-CT. The combined-modality sensitive reading and the sestamibi SPECT/CT had the highest ROC, which was not significantly different (*p* = 0.14) but significantly differed from that of 4D-CT (*p* = 0.0006). Sestamibi SPECT/CT had a significantly different (*p* = 0.0006) ROC curve that was greater than the ROC curve of the combined-modality-specific reading. There was no significant difference in the ROC curve between sestamibi SPECT/CT and 4D-CT (*p* = 0.11).

**Table 2:** Comparison of receiver operating characteristic curve

		*p* Value of Comparison	
Parameter	Performance ROC Estimate	Sestamibi SPECT/CT	4D-CT
Sestamibi SPECT/CT	0.90 (0.87, 0.92)	NA	0.11
4D-CT	0.88 (0.85, 0.90)	0.11	NA
Combined 4D-CT with Sestamibi SPECT/CT			
Sensitive reading	0.91 (0.89, 0.93)	0.14	0.0006
Specific reading	0.86 (0.83, 0.89)	0.0006	0.14
Note; NA, not available			

Table 3 summarizes the accuracy, sensitivity, specificity, PPV and NPV of all the modalities. Sestamibi SPECT/CT exhibited an accuracy of 92% (95% confidence interval: 90%, 94%; 891 of 968), similar to that of 4D-CT (91%; 95% confidence interval: 89%, 92%; 876 of 968), combined-modality sensitive reading (91%; 95% confidence interval: 89%, 93%; 878 of 968) and combined-modality specificity reading (92%; 95% confidence interval: 90%, 94%; 889 of 968). The sensitivity of combined-modality sensitive reading was higher than that of sestamibi SPECT/CT (sensitivity, combined-modality sensitive reading vs. sestamibi SPECT/CT: 92% [95% confidence interval: 88%, 95%; 230 of 251] vs. 85% [95% confidence interval: 80%, 89%; 231 of 251]). 4D-CT demonstrated sensitivity superior to that of combined-modality-specific reading (sensitivity, 4D-CT vs. combined-modality-specific reading: 81% [95% confidence interval: 76%, 86%; 204 of 251] vs. 75% [95% confidence interval: 69%, 80%; 287 of 251]). Combined-modality-specific readings were more specific than combined-modality-sensitive readings were (98% [95% confidence interval: 97%, 99%; 702 of 717 and 90% [95% confidence intervals: 88%, 93%; 648 of 717], respectively). Sestamibi SPECT/CT revealed a specificity of 95% (95% confidence interval: 93%, 96%; 678 of 717), similar to that of 4D-CT.

**Table 3:** Diagnostic performance in the patients with primary hyperparathyroidism

Parameter	Sestamibi SPECT/CT			4D-CT			Combined 4D-CT with Sestamibi SPECT/CT Sensitive Reading			Combined 4D-CT with Sestamibi SPECT/CT Specific Reading		
	Numerator/Denominator Estimate			Numerator/Denominator Estimate			Numerator/Denominator Estimate			Numerator/Denominator Estimate		
Overall												
Accuracy	891/968	0.92(0.90, 0.94)		876/968	0.91(0.89, 0.92)		878/968		0.91(0.89, 0.93	889/968	0.92(0.90, 0.94)	
Sensitivity	213/251	0.85 (0.80, 0.89)		204/251	0.81 (0.76, 0.86)		230/251		0.92(0.88, 0.95)	187/251	0.75 (0.69, 0.80)	
Specificity	678/717	0.95 (0.93, 0.96)		672/717	0.94 (0.92, 0.96)		648/717		0.90(0.88, 0.93)	702/717	0.98 (0.97, 0.99)	
PPV	213/252	0.85 (0.80, 0.89)		204/249	0.82 (0.77, 0.87)		230/299		0.77(0.72, 0.82)	187/202	0.93 (0.89, 0.96)	
NPV	678/716	0.95 (0.93, 0.96)		672/719	0.93 (0.92, 0.95)		648/669		0.97(0.96, 0.98)	702/766	0.92 (0.90, 0.93)	
Single gland disease												
Accuracy	860/928		0.93(0.91, 0.94)	842/928		0.91(0.89, 0.93)	844/928	0.91(0.89, 0.93)		858/928		0.92(0.91, 0.94)
Sensitivity	201/232		0.87(0.82, 0.91)	191/232		0.82(0.77, 0.87)	215/232	0.93(0.89, 0.96)		177/232		0.76(0.71, 0.82)
Specificity	659/696		0.95(0.93, 0.96)	651/696		0.94(0.91, 0.95)	629/696	0.90(0.88, 0.93)		681/696		0.98(0.97, 0.99)
PPV	201/238		0.84(0.80, 0.89)	191/236		0.81(0.76, 0.86)	215/282	0.76(0.71, 0.81)		177/192		0.92(0.88, 0.96)
NPV	659/690		0.96(0.94, 0.97)	651/692		0.94(0.92, 0.96)	629/646	0.97(0.96, 0.99)		681/736		0.92(0.90, 0.94)
Mutligland disease												
Accuracy	31/40		0.78(0.65, 0.90)	34/40		0.85(0.74, 0.96)	34/40	0.85(0.74, 0.96)		31/40		0.78(0.65, 0.90)
Sensitivity	12/19		0.63(0.41, 0.85)	6/19		0.32(0.11, 0.52)	15/19	0.79(0.60,0.97)		9/19		0.47(0.25, 0.70)
Specificity	19/21		0.90(0.78, 1.0)	21/21		1.00(1.00, 1.0)	19/21	0.90(0.78, 1.0)		21/21		1.00(1.00, 1.0)
PPV	12/14		0.86(0.67, 1.0)	13/13		1.00 (1.0, 1.0)	15/17	0.88(0.73, 1.0)		10/10		1.00(1.00, 1.0)
NPV	19/26		0.73(0.56, 0.90)	21/27		0.78(0.62, 0.93)	19/23	0.83(0.67, 0.98)		21/30		0.7(0.54, 0.86)
Note: data in parentheses are 95% confidence intervals.												

Similar trends were noted in the subset analysis of patients with single-gland disease: the accuracy of sestamibi SPECT/CT was 93% (95% confidence interval: 91%, 94%; 860 of 928), whereas it was 91% (95% confidence interval: 89%, 93%; 842 of 928) for 4D-CT and 92% (95% confidence interval: 91%, 94%; 858 of 928) for combined-modality-specific reading. Similar to the results for all patients, the sensitivity and specificity for all modalities in patients with single-gland diseases ranged from 76 to 93% and 90-98%, respectively.

**Fig. 3:**
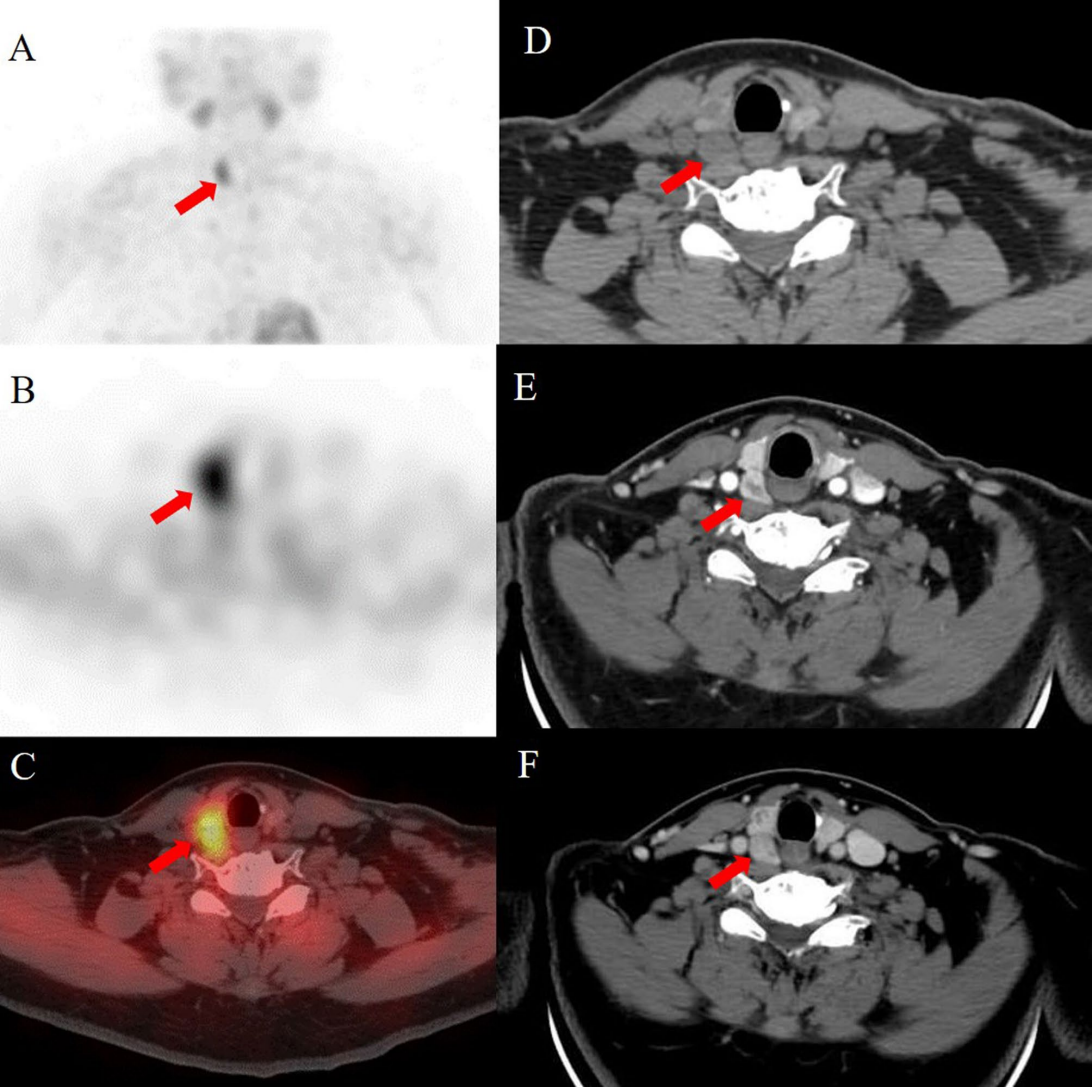
Images of a 53-year-old woman with primary hyperparathyroidism. Sestamibi SPECT/CT images (**A**, MIP SPECT; **B**, axial SPECT; **C**, fusion SPECT/CT) reveal focal radioactivity uptake localized to a single abnormal right superior parathyroid gland (red arrows). 4D-CT (**D**, axial noncontrast-enhanced; **E**, arterial phase; **F**, delayed phase) showing a vividly enhancing lesion at the arterial phase and washout of contrast material with decreasing attenuation compared with the atrial phase at the delayed phase (red arrows). The patient underwent parathyroidectomy for a single right superior parathyroid adenoma. Normal PTH and calcium levels were observed after 6 months of follow-up

**Fig. 4:**
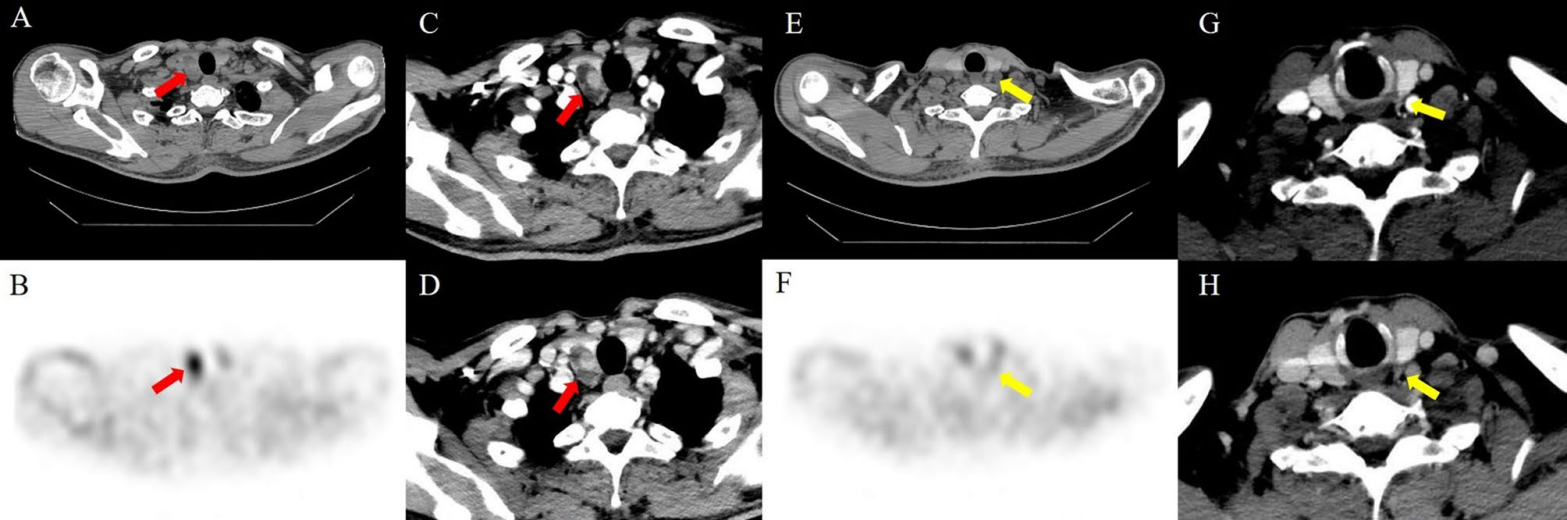
Image of a 57-year-old man with primary hyperparathyroidism. Axial noncontrast-enhanced CT (**A**) image showing an oval lesion (red arrows) localized to the right inferior parathyroid gland. Axial sestamibi SPECT image (**B**) showing focal radioactivity uptake localized to a single abnormal right superior parathyroid gland (red arrow). Axial 4D-CT (**C**, arterial phase; **D**, delayed phase) reveals an enhanced right inferior parathyroid gland (arrows) corresponding to the same gland identified via sestamibi SPECT/CT. Additional mild focal radioactivity uptake was noted in the left superior parathyroid gland (yellow arrows) on sestamibi SPECT/CT (**E**, axial noncontrast-enhanced; **F**, axial SPECT), which was not obscured on 4D-CT (**G**, arterial phase; **H**, delayed phase). The patient underwent bilateral four-gland exploration for left superior parathyroid hyperplasia and right inferior parathyroid adenoma. Normal PTH and calcium levels were observed after 19 months of follow-up

**Fig. 5:**
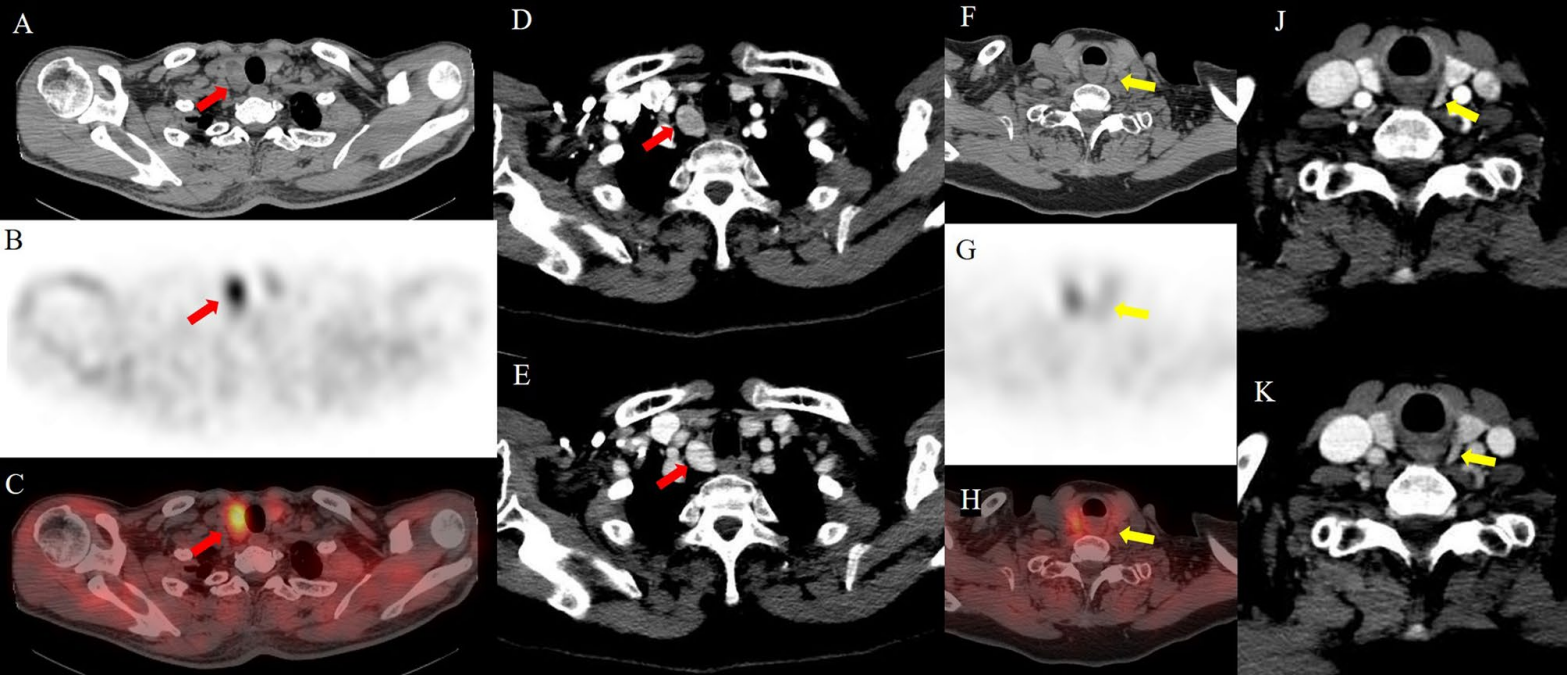
Image of an 83-year-old woman with primary hyperparathyroidism. Sestamibi SPECT/CT images (**A**, axial noncontrast-enhanced; **B**, axial SPECT; **C**, fusion SPECT/CT) reveal focal radioactivity uptake localized to a single abnormal right inferior parathyroid gland (red arrows). 4D-CT (**D**, arterial phase; **E**, delayed phase) shows a vividly enhancing lesion at the arterial phase and persistence of contrast material with increasing attenuation compared with the atrial phase at the delayed phase (red arrows). An additional left superior parathyroid gland (yellow arrows) was observed on noncontrast-enhanced CT (**F**), which revealed no focal uptake on sestamibi SPECT/CT (**G**, axial SPECT; **H**, fusion SPECT/CT), but mild moderate enhancement was noted on 4D-CT (**J**, arterial phase; **K**, delayed phase). The patient underwent bilateral four-gland exploration for left superior parathyroid hyperplasia and right inferior parathyroid adenoma. Normal PTH and calcium levels were observed after 26 months of follow-up

In patients with multigland disease, 4D-CT had a greater accuracy than sestamibi SPECT/CT (85% [95% confidence interval: 74%, 96%; 34 of 40] vs. 78% [95% confidence interval: 65%, 90%; 31 of 40], respectively) and was similar to the combined-modality sensitive reading (85%, 34 of 40). Compared with sestamibi SPECT/CT, combined-modality sensitive reading had greater sensitivity (79% [95% confidence interval: 60%, 97%; 15 of 19] vs. 63% [95% confidence interval: 41%, 85%; 12 of 19], respectively). Compared with 4D-CT, combined-modality-specific reading had greater sensitivity (47% [95% confidence interval: 25%, 70%; 9 of 19] vs. 32% [95% confidence interval: 11%, 52%; 6 of 19], respectively). Higher specificity was noted for the 4D-CT and combined-modality-specific readings (100%; 21 of 21]) than for the sestamibi SPECT/CT and combined-modality-sensitive readings (90%; 19 of 21). Figures 3, 4 and 5 present examples of the modalities used in patients with single-gland and multigland diseases.

## Discussion

We evaluated the diagnostic performance of sestamibi SPECT/CT and 4D-CT by using a combination of images and performed a head-to-head comparison of the two individual modalities in terms of preoperative localization in patients with PHPT at our institution. Our results revealed that the diagnostic performance of sestamibi SPECT/CT was similar to that of 4D-CT; this was indicated by similar ROC values (ROC, sestamibi SPECT/CT vs. 4D-CT, 0.90 vs. 0.88, respectively). Compared with 4D-CT, combined-modality sensitive reading (ROC, 0.91) improved performance. Sestamibi SPECT/CT showed similar accuracy to 4D-CT and combined-modality reading in patients with single-gland disease (93% vs. 91% vs. 91–92%, respectively), but 4D-CT had a greater accuracy than sestamibi SPECT/CT in patients with multigland disease (85% vs. 78%).

Most previous studies compared sestamibi SPECT/CT and 4D-CT for preoperative localization and analyzed the sensitivity and accuracy per quadrant. In a large retrospective study on the diagnostic performance of 4D-CT and sestamibi SPECT/CT in 400 patients by Yeh et al. [12], 4D-CT had higher sensitivity than sestamibi SPECT/CT in patients with single and multigland disease (92.5% vs. 75.1% and 58.2% vs. 30.8%, respectively.) Our study similarly demonstrated that sestamibi SPECT/CT and 4D-CT had greater sensitivities for single diseases than for multiple diseases (87%, 82% and 63%, 32%, respectively). The higher sensitivity of sestamibi SPECT/CT in our study is likely because there were fewer patients with multigland disease (3.7% vs. 19.8%) who were subjected to the same sestamibi SPECT/CT acquisition protocol. Wan et al. [13] reported results similar to those of our study in 911 patients, with a sensitivity of 85% for 4D-CT compared with 81% in our study. The sensitivity of sestamibi SPECT/CT (85%) was also greater than that reported by Wan et al. (68%). The possible explanations are that the sestamibi protocols vary among different studies, patient cohorts across various studies and different image interpretation criteria, which can influence diagnostic performance. Several studies have emphasized and provided a comprehensive overview of the challenges associated with different sestamibi imaging protocols [14, 15]. Recently, a meta-analysis including 23 studies with 4695 patients revealed that the pooled accuracy, sensitivity and specificity were greater with 4D-CT (87.7%, 79.9%, and 84.5%, respectively) than with sestamibi SPECT/CT (70.6%, 64.0%, and 78.9%, respectively) [16]. Kedarisetty et al. compared SPECT/CT with 4D-CT and reported their sensitivity (77% vs. 80%), specificity (71% vs. 75%), and accuracy (77% vs. 79%) in 58 patients with PHPT [17]. Our study revealed the superiority of the diagnostic performance of 4D-CT and sestamibi SPECT/CT. Notably, only 14 of 58 patients underwent 4D-CT, and all patients underwent preoperative sestamibi SPECT/CT with 740 MBq sestamibi.

In our study, 242 patients with 968 glands were included in a lesion-based analysis to compare the diagnostic performance of sestamibi SPECT/CT and 4D-CT in preoperative localization. Our study differs from previous studies because we utilized pathohistological reports and follow-up data as the standards for evaluating imaging outcomes. Different medical specialists reevaluated the imaging modalities for localization and were blinded to the pathohistological and other imaging results.

Our study had several limitations. First, the study was retrospective and performed at a single institution, thus there are inherent limitations. Some patients with negative or inconclusive imaging findings may not have undergone parathyroidectomy or were excluded from the analysis, especially patients with multigland disease. This limitation indicates that the diagnostic value may be overestimated in our study. Second, during the study, the choice of imaging method depends on several factors, including surgeon preference, radiologist expertise and patient history of allergy to contrast agents used for CT and renal function. Sestamibi SPECT/CT is a common preoperative localization method at our institution, and we have accumulated rich experience; however, 4D-CT has not yet been widely applied. Finally, 4D-CT may be performed after the results of imaging examinations such as ultrasonagraphy or sestamibi SPECT/CT are negative or inconclusive. These potential bias factors should be addressed in the future in a prospective, multicenter study and direct head-to-head comparison studies.

## Conclusions

The results of our study indicate that sestamibi SPECT/CT had high accuracy in preoperative localization in patients with PHPT. Compared with sestamibi SPECT/CT alone, 4D-CT and combined-modality reading did not improve diagnostic performance. More research, ideally with standardized imaging protocols and uniform diagnostic criteria, is needed to determine the clinical significance of preoperative localization.

## Data Availability

No datasets were generated or analysed during the current study.

## Abbreviations

PHPT: Primary hyperparathyroidism
4D-CT: Four-dimensional Computed tomography
PTH: Parathyroid hormone
Sestamibi SPECT/CT: Sestamibi single-photon emission computed tomography/computed tomography
PPV: Positive predictive value
NPV: Negative predictive value
ROC: Receiver operating characteristic
